# Mortality in nursing home residents: A longitudinal study over three years

**DOI:** 10.1371/journal.pone.0203480

**Published:** 2018-09-18

**Authors:** Corinna Vossius, Geir Selbæk, Jurate Šaltytė Benth, Sverre Bergh

**Affiliations:** 1 Centre for Age-related Medicine, Stavanger University Hospital, Stavanger, Norway; 2 Centre for Old Age Psychiatry Research, Innlandet Hospital Trust, Brumunddal, Norway; 3 Norwegian National Advisory Unit on Ageing and Health, Vestfold Hospital Trust, Tønsberg, Norway; 4 Institute of Health and Society, University of Oslo, Oslo, Norway; 5 Institute of Clinical Medicine, University of Oslo, Oslo, Norway; 6 Health Services Research Unit, Akershus University Hospital, Lørenskog, Norway; Cardiff University, UNITED KINGDOM

## Abstract

**Objective:**

Nursing home (NH) stay is the highest level of formal care. With the expected demographic changes ahead, the need for NH placement will put an increasing socioeconomic strain on the society. Survival in NHs and factors predicting survival are important knowledge in order to evaluate NH admission policies and plan future NH capacity.

**Methods:**

We followed 690 NH residents included at admission to NH over a period of three years. Participants were examined at baseline (BL) and every six months. Demographic and clinical data were collected, including comorbidity, severity of cognitive impairment, dependency in activities of daily living (ADL) and neuropsychiatric symptoms. Median survival was calculated by the Kaplan-Meier analysis, and factors associated with mortality were identified by Cox models with baseline and time-dependent covariates.

**Results:**

Median survival in NH was 2.2 years (95% confidence interval [CI]: 1.9–2.4). Yearly mortality rate throughout the three-year observation period was 31.8%. Mortality was associated with higher age and comorbidity at BL, and more severe dementia, higher ADL-dependency, less severe psychotic symptoms, and a lower BMI throughout the study period. Of the organizational variables, living on a ward with more residents resulted in a higher risk of mortality.

**Conclusion:**

In conclusion, the NH mortality rate remained stable throughout the three-year study period with about one third of the residents deceasing each year. Individual resident characteristics appeared to be more important than organizational variables for predicting mortality risk. The finding of an association between ward size and mortality risk deserves further investigation in future studies.

## Introduction

Population projections predict an increase in the share of elderly in developed countries, and thereby an increase in the number of frail persons in need of informal and formal care. In Norway, the highest level of formal care is nursing home (NH) stay, where care and supervision are provided on a 24/7 basis. Projections estimate that due to demographic trends the number of nursing home beds will have to be increased by 50% by 2030 if the criteria for NH admission and the levels of home care remain unchanged [1].

Mortality in NH residents and the patient characteristics or environmental factors at the NHs associated to mortality are important figures for health authorities and decision makers in order to estimate future need for NH capacity and survey the effect of NH admission policies over time. A previous Norwegian study found a median survival time of 2.1 years, while in other studies median survival was 2.3 years in an Irish cohort and a US cohort, and 2.6 years in an Icelandic cohort [2–5]. Studies with shorter follow-up have reported one-year mortality rates between 17.4% and 35.0% [6–8]. Higher age, male gender, low functioning in activities of daily living (ADL), poorer physical health, and low nutrition status are patient characteristics associated with mortality [3, 5, 6, 9,10].

The project Resource Use and Disease Course in Dementia—Nursing Home (REDIC-NH) included 696 persons at admission to NH. They were followed over a period of 36 months or until death, with clinical examinations at baseline (BL) and every six months thereafter. The aim of this study was to evaluate mortality rates in Norwegian NH residents over time and to identify the demographic, clinical and organizational factors associated with mortality.

## Material and methods

The REDIC-NH study is an observational longitudinal study including patients from a convenience sample of 47 NHs in four Norwegian counties, representing small and large NHs located in urban and rural areas [11]. Inclusion was at admission to the NH, with residents followed over an observation period of 36 months or until death. Inclusion took place between January 2012 and August 2014. Four NHs withdrew from the study during the observation period.

Inclusion criteria were: the participant should be 65 years of age or older or have dementia irrespective of age. In addition, expected survival should be six weeks or more. Only residents that completed the baseline (BL) assessment were included in the study. BL assessment was aimed to be completed within four weeks after inclusion, but the mean interval between admission and the completed BL assessment was 10.5 weeks (SD10.6). The participants were monitored with a clinical follow-up (FU) every six months, at FU6, FU12, FU18, FU24, FU30 and FU36.

Data collection was performed by trained healthcare workers at the NH, mainly registered nurses (74%) under supervision of 10 research nurses. The research nurses completed a five-day training prior to study start, while the data collectors completed a two-day training. Data were collected through structured interviews with the patient and a caregiver.

Organizational data about the NHs were collected at the beginning of the study period. As about 26% of the residents moved to a new ward at least once during the NH stay, we chose to analyse the impact of organizational variables on mortality with data from the ward where the patients stayed at the last FU examination before study end or death.

All rating scales and inventories were applied in established Norwegian versions applied in a number of previous studies [12–19]. The following demographic and clinical data were collected:

Demographic data, including gender, age, and living status before admission to NH, were collected by reviewing the patient’s journal.

Diagnosis of dementia at BL was set according to the criteria of ICD-10 [20]. The diagnosis of dementia was set independently by two of the authors (SB and GS), both specialists in psychiatry and experienced in old age psychiatry and research, based on all available information about the participants. When no consensus was reached, a third psychiatrist was consulted.

Clinical Dementia Rating Scale (CDR) was applied to assess the severity of dementia. The rating scale comprises six items [12,13,14]. For statistical purposes we calculated the CDR-sum of boxes (CDR-SOB) that offers an extended range of values and is calculated by adding the item scores (range 0–18), where higher scores indicate more severe dementia [21].

Neuropsychiatric Inventory nursing home version (NPI-NH) assesses neuropsychiatric symptoms. The instrument contains 12 items and is conducted as an interview with a caregiver. Severity (scored 0–3) was multiplied by frequency (scored 0–4), giving an item score from 0–12, where higher scores indicate more severe symptoms [15, 16]. Based on a previous principal component analysis, we created the following sub-syndromes: NPI-Agitation (agitation/aggression, disinhibition and irritability), NPI-Psychosis (delusions and hallucinations) and NPI-Affective (depression and anxiety) [11].

Physical Self-Maintenance Scale (PSMS) consists of six items (scored 1–5) and assesses personal activities of daily living (PADL) function. The overall score ranges from 6 to 30, where higher scores indicate higher PADL dependency [17].

General Medical Health Rating (GMHR) rates physical health. It consists of one item, with the four categories excellent, good, fair or poor [18].

Charlson’s comorbidity index was applied to establish comorbidity at BL [19].

Body mass index (BMI) relates a person’s weight to her height.

The following organizational data were collected at the last FU examination before study end or death: number of inhabitants in the municipality, number of beds in the nursing home, type of ward (general ward, special care unit [SCU]), short-time stay, other), number of residents at the ward, staff/resident ratio at the ward during daytime and evening shift, physician time (expressed as minutes per patient and week) and number of times a resident moved from one ward to another related to the time of observation.

The staff/resident ratio was calculated by applying the following formula:

$$\frac{5*(number\;of\;staff\;daytime+evening\;weekdays)+2*(number\;of\;staff\;daytime+evening\;weekend)}{Number\;of\;residents\;on\;the\;ward}$$

### Statistics

Demographic factors and clinical symptoms were described by means and standard deviations (SD) or frequencies and percentages. The group differences were analysed by Student’s t-test for continuous variables and by χ^2^-test for categorical variables. Missing values for PSMS items were imputed for cases with fewer than 50% missing among all items by generating an empirical distribution based on non-missing cases for each item and drawing a random number from it. Kaplan-Meier analysis was performed to estimate median survival time. Mortality rates were calculated by dividing the number of participants dying by the number of person-years in 6-month and 1-year periods. A Cox regression model was estimated to assess the impact of demographic variables (measured at baseline), and clinical and organizational variables (time-dependent and measured at baseline only) on mortality. Ward level was entered into the model as random effect to control for possible intra-ward correlations. Bivariate and multiple models were estimated. The multiple model was further reduced by applying Akaike’s Information Criteria (AIC), where the smaller value means a better model.

Results with p-values below 0.05 were considered statistically significant. The analyses were performed in SPSS v25 and SAS v9.4.

### Ethics

The resident’s capacity to consent in the study was considered by the NH staff, including the physician. Written informed consent was obtained from those participants with full capacity to consent or from the next-of-kin on behalf of the participant in case of reduced capacity to consent. The Regional Committee for Medical Research Ethics–South East Norway approved the study (2011/1378a).

## Results

### Study sample

**Fig 1:**
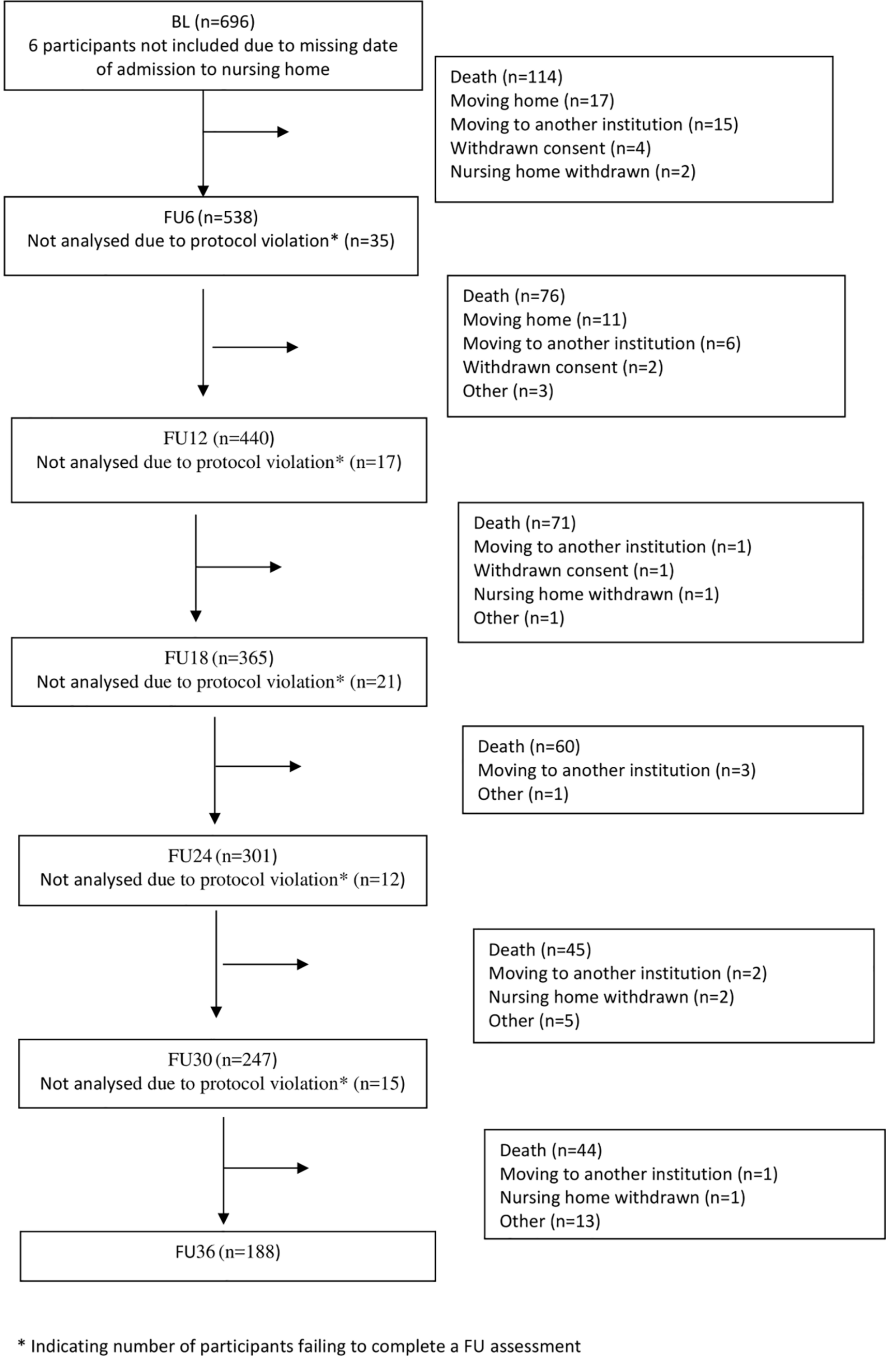
Flow chart. Flow chart describing attrition from baseline (BL) to 36 months follow-up (FU36).

A total of 696 participants were included in the REDIC-NH study. We included 690 participants where the date of admission to NH could be established. Mean age of the participants was 84.4 years (range 49 to 105, SD = 7.5), 63.9% were female, and 83.9% had dementia at BL. Table 1 shows demographic and clinical characteristics at BL and organizational characteristics at the last FU carried out for the whole study sample and according to whether or not the participant died during the observation period. At FU36, 188 participants (27.2%) were still in the study, while 410 participants (59.4%) had died and 92 participants (13.3%) had dropped out for various reasons. Fig 1 presents a flow chart describing attrition from BL to FU36. In addition, the number of participants failing to complete an FU assessment is indicated as “Not analysed due to protocol violation”.

**Table 1:** Demographic and clinical characteristics at BL and organizational characteristics at the last FU carried out for the whole study population and according to whether the participant survived until the end of the study or dropped out, or died during the observation period.

	All participants (n = 690)	Alive at FU36 or dropped out during observation period (n = 280)	Deceased during observation period (n = 410)	*p*-value
Observation period in years, median (min/max)	1.9 (0.1/3.9)	3.0 (0.1/3.9)	1.1 (0.7/3.8)	<0.001
**Demographic and clinical variables at BL**				
Age, mean (SD)	84.4 (7.5)	82.5 (8.0)	85.7 (6.8)	<0.001
Gender, female (%)	441 (64)	191 (68)	250 (61)	0.052
Living with partner before admission (%)	207 (30)	80 (29)	127 (31)	0.522
Dementia (%)	574 (84)	234 (85)	340 (84)	0.797
CDR-SOB, mean (SD)	10.3 (4.3)	9.9 (4.2)	10.5 (4.4)	0.078
PSMS, mean (SD)	15.3 (4.5)	14.3 (4.4)	16.0 (4.5)	<0.001
NPI-sum, mean (SD)	13.7 (16.5)	13.1 (16.3)	14.1 (16.6)	0.446
NPI-sub-syndromes, mean (SD):				
Agitation	4.1 (7.0)	4.1 (7.3)	4.0 (6.8)	0.927
Psychosis	1.7 (3.9)	1.5 (3.7)	1.8 (4.0)	0.458
Affective	3.7 (6.0)	3.5 (5.4)	3.8 (5.9)	0.441
GMHR (%)				
Poor or fair	345 (52)	117 (43)	228 (58)	<0.001
Charlson’s comorbidity index, mean (SD)	3.0 (2.4)	2.4 (1.8)	3.3 (2.7)	<0.001
BMI, mean (SD)	23.9 (4.5)	24.4 (4.4)	23.6 (4.5)	0.041
**Organizational variables at last FU carried out**				
Inhabitants of municipality, mean (SD)	49 484 (91 556)	55 448 (98 168)	45 440 (86 593)	0.166
Number of beds in NH, mean (SD)	75.8 (43.4)	74.5 (41.8)	76.7 (44.4)	0.516
Type of ward (%)				0.383
Short-time stay	72 (10)	33 (12)	39 (10)	
General	393 (57)	151 (54)	245 (60)	
SCU	217 (31)	93 (33)	124 (30)	
Other	5 (1)	3 (1)	2 (1)	
Number of residents on ward, mean (SD)	12.3 (7.0)	11.8 (7.4)	12.6 (6.7)	0.156
Staff/resident ratio, mean (SD)	3.7 (1.1)	3.7 (1.1)	3.6 (1.0)	0.267
Times moved per observation time, mean (SD)	0.19 (0.52)	0.21 (0.61)	0.17 (0.45)	0.256

BL = baseline; FU = follow-up; SD = standard deviation; CDR-SOB = Clinical dementia rating scale–sum of boxes; PSMS = Physical self-maintenance scale; NPI = Neuropsychiatric inventory; NPI-Agitation = NPI sub-syndromes agitation/aggression, disinhibition and irritability, NPI-Psychosis = NPI sub-syndromes delusions and hallucinations; NPI-Affective = NPI sub-syndromes depression and anxiety; GMHR = General medical health rating; BMI = Body mass index; NH = nursing home; SCU = special care unit.

### Mortality rates and mean survival

**Fig 2:**
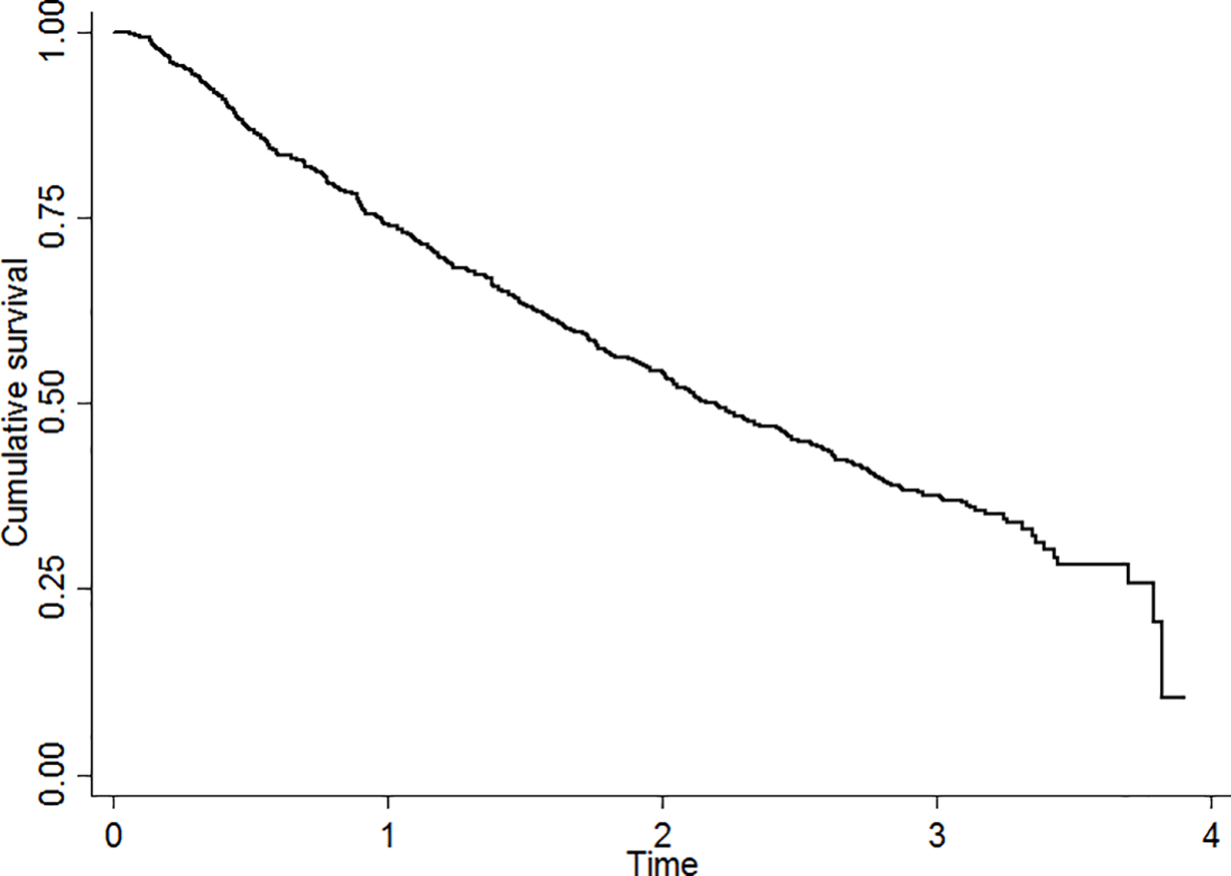
Kaplan-Meier analysis of survival for the whole study population.

Mortality rates between the 6-month FU examinations shown in Table 2, varied between 14.4% (FU6 to FU12) and 18.4% (FU30 to FU36). The mortality rate throughout the observation period is also illustrated in the Kaplan-Meier analysis as shown in Fig 2.

**Table 2:** Number of participants and number of deceased persons during the study period.

	BL	FU6	FU12	FU18	FU24	FU30	FU36
Participants	690	537	440	365	301	247	188
Deceased (%)		114 (17.0)	76 (14.4)	71 (16.2)	60 (16.5)	45 (15.2)	44 (18.4)

BL = baseline; FU = follow-up.

The yearly mortality rate throughout the observation period was 31.8., while the median survival was 2.2 years (95% confidence interval (CI) 1.9–2.4 years).

### Demographic, clinical and organizational variables related to mortality

Table 3 shows the results of the bivariate Cox regression models, the multiple model with all covariates included, and the AIC-reduced multiple model. Higher age, higher comorbidity, more severe dementia, higher PADL-dependency, less severe psychotic symptoms, and a lower BMI were associated with higher mortality. Of the organizational variables, living on a ward with a more residents resulted in a higher risk of mortality.

**Table 3:** Results of the Cox model.

Variable	Unadjusted model		Adjusted model		Adjusted model, AIC-reduced	
	HR (95% CI)	p-value	HR (95% CI)	p-value	HR (95% CI)	p-value
***Patient characteristics***						
Age	1.03 (1.01; 1.05)	**0.004**	1.04 (1.01; 1.06)	**0.003**	1.04 (1.01; 1.06)	**0.002**
Gender						
- Female	1		1			
- Male	1.10 (0.81; 1.49)	0.563	1.16 (0.82; 1.64)	0.399		
Living alone						
- No	1		1			
- Yes	0.91 (0.66; 1.25)	0.545	0.83 (0.58; 1.19)	0.315		
Dementia at BL						
- No	1		1	0.915		
- Yes	1.22 (0.75; 1.97)	0.425	0.97 (0.55; 1.72)			
Charlson’s comorbidity index at BL	1.11 (1.04; 1.18)	**0.001**	1.13 (1.06; 1.21)	**<0.001**	1.13 (1.06; 1.21)	**<0.001**
*Time-dependent*						
CDR-SOB	1.08 (1.03; 1.12)	**<0.001**	1.07 (1.01; 1.14)	**0.022**	1.05 (1.00; 1.11)	**0.040**
PSMS	1.09 (1.06; 1.13)	**<0.001**	1.08 (1.03; 1.13)	**0.001**	1.07 (1.03; 1.12)	**0.001**
NPI-Affective	1.02 (0.99; 1.04)	0.172	1.04 (1.01; 1.07)	**0.014**	1.03 (1.00; 1.05)	0.051
NPI-Psychosis	0.97 (0.93; 1.01)	0.102	0.95 (0.90; 0.99)	**0.040**	0.94 (0.89; 0.98)	**0.005**
NPI-Agitation	0.98 (0.96; 1.01)	0.173	0.99 (0.96; 1.02)	0.544		
GMHR						
- Poor/fair	1.21 (0.88; 1.67)	0.244	0.79 (0.56; 1.12)			
- Good/excellent	1		1	0.192		
BMI	0.94 (0.91; 0.98)	**0.001**	0.96 (0.93; 0.99)	**0.032**	0.96 (0.93; 0.99)	**0.030**
***Organizational variables***						
Inhabitants in municipality	1.00 (1.00; 1.00)	0.256	1.00 (1.00; 1.00)	0.115		
Beds in NH	1.00 (0.99; 1.01)	0.170	1.00 (0.99; 1.01)	0.498		
Times moved	1.11 (0.80; 1.55)	0.538	0.91 (0.63; 1.33)	0.635		
Type of ward						
- General	1		1			
- SCU	0.81 (0.58; 1.13)	0.217	0.89 (0.61; 1.31)	0.570		
- Short-time	0.81 (0.44; 1.48)	0.488	0.82 (0.44; 1.53)	0.530		
- Other	0.66 (0.16; 2.68)	0.559	0.67 (0.16; 2.74)	0.572		
Patients on ward	1.04 (1.01; 1.06)	**0.002**	1.03 (1.00; 1.06)	**0.031**	1.03 (1.01; 1.05)	**0.016**
Staff/ resident ratio	0.96 (0.83; 1.11)	0.586	0.99 (0.86; 1.16)	0.992		

AIC = Akaike’s Information Criteria; HR = Hazard ratio; CI = Confidence interval; BL = baseline; CDR-SOB = Clinical dementia rating scale–sum of boxes; PSMS = Physical self-maintenance scale; NPI = Neuropsychiatric inventory; NPI-Agitation = NPI sub-syndromes agitation/aggression, disinhibition and irritability, NPI-Psychosis = NPI sub-syndromes delusions and hallucinations; NPI-Affective = NPI sub-syndromes depression and anxiety; GMHR = General medical health rating; BMI = Body mass index; NH = nursing home, SCU = special care unit.

## Discussion

The REDIC-NH study comprises 696 participants, of which 690 were included in this longitudinal study that assessed mortality rates and factors associated with mortality. We found that median survival was 2.2 years, while the yearly mortality rate was 31.8%. Factors associated with mortality were higher age, higher comorbidity, more severe dementia, higher PADL-dependency, less severe psychotic symptoms, and a lower BMI. Of the organizational variables, living on a ward with more residents resulted in a higher risk of mortality.

Mortality was predicted by reduced overall health as indicated by more severe dementia and higher comorbidity, and a higher PADL-dependency. As in previous studies, we found that higher age was associated with higher mortality. However, contrary to previous studies, we found no gender differences [5, 8, 22]. Likewise, our finding that more severe psychosis was associated with a lower mortality risk is in contradiction to previous findings [23]. As psychosis was a predictor for NH admission in a Norwegian study [24], these residents might be younger and have better somatic health as confounding factors regarding mortality. Lower BMI was related to higher mortality, indicating that weight loss may be part of the pre-terminal stage in a person’s life [9, 10]. We also found that wards with more residents had higher mortality rates. This is probably due to the fact that small wards are often SCUs, with a special focus on persons with dementia and neuropsychiatric symptoms, while residents where physical diseases and ADL-dependency are the main reasons for admission, live in larger general wards [1]. Worse somatic health and higher ADL-dependency may again explain our finding of higher mortality on these wards.

There are few studies evaluating mortality in NH residents, and results from different health systems might be difficult to compare to each other due to varying criteria for admission to an NH. However, studies from the US, Ireland and Iceland report median survival times between 2.3 and 2.8 years, which are higher but still in the same range as our finding of 2.2 years. [3–5; 8; ]. The reported annual mortality rates in two US studies match our finding of 31.8%, with 25.6% and 35.0%, respectively [7, 8]. However, a French study reports a significantly lower mortality rate of 17.4% [6]. Median survival time is a marker for changes in admission policies over time, reflecting the severity of the patients’ overall frailty. A register-based Norwegian study conducted between 1994 and 2004 found a median survival of 2.1 years, as compared to 2.2 years in the present study, indicating similar NH admission criteria despite an important health care reform in 2012 that is said to result in NH residents with a higher morbidity than before [2].

### Strengths and limitations of the study

We followed a large cohort of 690 participants from 47 NHs in a longitudinal design over three years, with clinical examinations twice a year. High quality of the data collection was secured by a standardized interview carried out by healthcare workers with adequate training under the supervision of research nurses. Furthermore, the Norwegian health and social system provides a rather homogenous environment for health service research as there are hardly any private actors on the market. Almost all care services are rendered by the municipalities with comparable criteria for admission of residents, staff/resident ratios, medical services and refund systems.

The main weakness of the study is that our sample might not be representative of the general NH population in Norway. Only participants that completed the BL examination were included, and mean time from admission to BL was 10.5 weeks. We thus excluded some participants who were eligible for the study but who died shortly after admission to NH. In a former register-based study, we found that 7% of NH residents died within the first three months of their NH stay [1]. Thus, the mortality rate for the first year and hence the overall mortality rate will be underestimated, and a median survival at NH with 2.2 years is consequently slightly overestimated. In addition, many of the NHs eligible for this study did not choose to participate, and not all patients eligible were included in the study. There might be a bias between residents willing to participate in the study and those who declined. As shown in a previous study only 38 of the 47 participating NHs collected data about age and gender of the residents eligible for inclusion but not consenting to participate. Of 1331 eligible residents in these 38 NHs, 607 were included and 724 excluded (205 declined inclusion, 191 died before inclusion took place, and 328 for reasons unknown). Residents included were slightly older (84.5 versus 83.6 years, p = 0.048), and there were also more women in the included sample compared to the group who did not participate (64.4% versus 56.6%, p = 0.004) [11]. Inter-rater reliability was not established and may thus have led to reduced data quality. However, the data collectors were under supervision of research nurses throughout the whole study period to diminish this effect. Moreover, the study sample included participants admitted to short-term as well as long-term wards, and this inclusion protocol may have resulted in a more heterogeneous study sample and a higher number of dropouts due to residents moving home again.

## Conclusion

In conclusion, the NH mortality rate remained stable throughout the three-year study period with about one third of the residents deceasing each year. Individual resident characteristics appeared to be more important than organizational variables for predicting mortality risk. The finding of an association between ward size and mortality risk deserves further investigation in future studies.

## References

[1] Vossius C, Selbæk G, Ydstebo A, Benth JS, Godager G, Luraas H. Ressursbruk og sykdomsforløp ved demens (REDIC). Sykehuset Innlandet, Report. 2015:1–155.

[2] Vossius C, Nilsen O, Larsen J. Parkinson’s disease and nursing home placement: the economic impact of the need for care. European journal of neurology. 2009;16(2):194–200. 10.1111/j.1468-1331.2008.02380.x 19146640

[3] McCann M, O'Reilly D, Cardwell C. A census-based longitudinal study of variations in survival amongst residents of nursing and residential homes in Northern Ireland. Age and ageing. 2009;38(6):711–7. Epub 2009/09/16. 10.1093/ageing/afp173 19752201

[4] Wieland D, Boland R, Baskins J, Kinosian B. Five-year survival in a program of all-inclusive care for elderly compared with alternative institutional and home- and community-based care. The journals of gerontology Series A, Biological sciences and medical sciences. 2010;65(7):721–6. Epub 2010/04/01. 10.1093/gerona/glq040 20354065

[5] Hjaltadottir I, Hallberg IR, Ekwall AK, Nyberg P. Predicting mortality of residents at admission to nursing home: a longitudinal cohort study. BMC Health Serv Res. 2011;11:86 Epub 2011/04/22. 10.1186/1472-6963-11-86 21507213 PMC3112069

[6] Tabue-Teguo M, Kelaiditi E, Demougeot L, Dartigues JF, Vellas B, Cesari M. Frailty index and mortality in nursing home residents in France: Results from the INCUR Study. J Am Med Dir Assoc. 2015;16(7):603–6. Epub 2015/03/15. 10.1016/j.jamda.2015.02.002 25769962

[7] Li S, Middleton A, Ottenbacher KJ, Goodwin JS. Trajectories over the first year of long-term care nursing home residence. J Am Med Dir Assoc. 2018;19(4):333–41. Epub 2017/11/08. 10.1016/j.jamda.2017.09.021 29108886 PMC6432771

[8] Gambassi G, Landi F, Lapane KL, Sgadari A, Mor V, Bernabei R. Predictors of mortality in patients with Alzheimer’s disease living in nursing homes. Journal of Neurology, Neurosurgery & Psychiatry. 1999;67(1):59–65. 10.1136/jnnp.67.1.59 PMC1736445 10369823

[9] Cereda E, Pedrolli C, Zagami A, Vanotti A, Piffer S, Opizzi A, et al Body mass index and mortality in institutionalized elderly. J Am Med Dir Assoc. 2011;12(3):174–8. Epub 2011/02/22. 10.1016/j.jamda.2010.11.013 21333917

[10] Veronese N, De Rui M, Toffanello ED, De Ronch I, Perissinotto E, Bolzetta F, et al Body mass index as a predictor of all-cause mortality in nursing home residents during a 5-year follow-up. J Am Med Dir Assoc. 2013;14(1):53–7. Epub 2012/11/13. 10.1016/j.jamda.2012.09.014 23141123

[11] Røen I, Selbæk G, Kirkevold Ø, Engedal K, Testad I, Bergh S. Resource use and disease course in dementia-nursing home (REDIC-NH): a longitudinal cohort study; design and patient characteristics at admission to Norwegian nursing homes. BMC health services research. 2017;17(1):365 10.1186/s12913-017-2289-x 28532443 PMC5441072

[12] Hughes CP, Berg L, Danziger WL, Coben LA, Martin R. A new clinical scale for the staging of dementia. British journal of psychiatry. 1982;140(6):566–72. 7104545 10.1192/bjp.140.6.566

[13] [cited 24.7.2018] Washington University in St Loius. Clinical Dementia Rating Assessment Protocol, Norwegian version. https://knightadrc.wustl.edu/cdr/PDFs/Translations/Norwegian%20Norway.pdf

[14] Selbaek G, Kirgevold Ø, Engedal K. The orevalence of psychiatric symptoms and behavioural disturbances and the use of psychotropic drugs in Norwegian Nursing Homes. International Jornal of Geriatric Psychiatry. 2007 22 (9): 843–9 10.1002/gps.1749 17193341

[15] Cummings JL, Mega M, Gray K, Rosenberg-Thompson S, Carusi DA, Gornbein J. The Neuropsychiatric Inventory comprehensive assessment of psychopathology in dementia. Neurology. 1994;44(12):2308–. 7991117 10.1212/wnl.44.12.2308

[16] Selbaek G, Kirkevold O, Sommer OH, Engedal K. The reliability and validity of the Norwegian version of the Neuropsychiatric Inventory, nursing home version (NPI-NH). Int Psychogeriatr. 2008;20(2):375–82 10.1017/S1041610207005601 17559707

[17] Lawton MP, Brody EM. Assessment of older people: self-maintaining and instrumental activities of daily living. Nursing Research. 1970;19(3):278. 5349366

[18] Lyketsos CG, Galik E, Steele C, Steinberg M, Rosenblatt A, Warren A, et al The General Medical Health Rating: a bedside global rating of medical comorbidity in patients with dementia. Journal of the American Geriatrics Society. 1999;47(4):487–91. 10203127 10.1111/j.1532-5415.1999.tb07245.x

[19] Charlson ME, Pompei P, Ales KL, MacKenzie CR. A new method of classifying prognostic comorbidity in longitudinal studies: development and validation. Journal of chronic diseases. 1987;40(5):373–83. 3558716 10.1016/0021-9681(87)90171-8

[20] Organization WHO. The ICD-10 classification of mental and behavioural disorders: clinical descriptions and diagnostic guidelines World Health Organization; 1992.

[21] O’Bryant SE, Waring SC, Cullum CM, Hall J, Lacritz L, Massman PJ, et al Staging dementia using Clinical Dementia Rating Scale Sum of Boxes scores: a Texas Alzheimer's research consortium study. Archives of neurology. 2008;65(8):1091–5. 10.1001/archneur.65.8.1091 18695059 PMC3409562

[22] Dale MC, Burns A, Panter L, Morris J. Factors affecting survival of elderly nursing home residents. International journal of geriatric psychiatry. 2001;16(1):70–6. Epub 2001/02/17. 11180488 10.1002/1099-1166(200101)16:1<70::aid-gps277>3.0.co;2-6

[23] Cohen-Mansfield J, Marx MS, Lipson S, Werner P. Predictors of mortality in nursing home residents. Journal of clinical epidemiology. 1999;52(4):273–80. 10235167 10.1016/s0895-4356(98)00156-5

[24] Wergeland JN, Selbæk G, Bergh S, Soederhamn U, Kirkevold Ø. Predictors of nursing home admission and death among community-dwelling people 70 years and older who receive domilicary care. Dement Geriatr Cogn Dis Extra. 2015 9 4;5(3):320–9. 10.1159/000437382 eCollection 2015 Sep-Dec. 26483831 PMC4608662

